# Optimal Placement of Irradiation Sources in the Planning of Radiotherapy: Mathematical Models and Methods of Solving

**DOI:** 10.1155/2015/142987

**Published:** 2015-10-12

**Authors:** Oleg Blyuss, Larysa Koriashkina, Elena Kiseleva, Robert Molchanov

**Affiliations:** ^1^Institute for Women's Health, University College London, London WC1E 6BT, UK; ^2^Department of Computational Mathematics and Mathematical Cybernetics, Faculty of Applied Mathematics, Oles Honchar Dnipropetrovsk National University, Dnipropetrovsk 49050, Ukraine; ^3^Department of System Analysis, Faculty of Information Technologies, State Higher Educational Institution “National Mining University”, Dnipropetrovsk 49050, Ukraine; ^4^SE “Dnepropetrovsk Medical Academy of Health Ministry of Ukraine”, Dnipropetrovsk 49044, Ukraine

## Abstract

This paper proposes and analyses a mathematical model for the problem of distribution of a finite number of irradiation sources during radiotherapy in continuous environments to maximize the minimal cumulative effects. A new algorithm based on nondifferentiable optimization techniques has been developed to solve this problem.

## 1. Introduction

The present work is devoted to the mathematical modelling of optimization problems arising in the planning of radiation therapy. Radiation therapy is a local-regional treatment of malignant tumors, with the main advantage being the possibility of wider local antitumor effects before surgery. Up to 70% of cancer patients undergo radiation treatment as an independent method or as a component of combination treatment (combination with surgery, chemotherapy) [1, 2].

In modern radiotherapy, many different types of ionizing radiation, which differ in biological effect, penetrability, and distribution of energy in radiation beam, are used as antitumor agents. Ionizing radiation must be supplied to the tumor strictly in certain doses, fractions, and time intervals and in certain places. The extent of exposure is required to include not only the primary tumor but also zones of subclinical spread of the tumor into the surrounding normal tissues, including lymph nodes. The main objective of radiation therapy is to bring a full dose to the tumor in an optimal way with more than 90% of patients with tumors of this localization and histological structure to be cured. In addition, normal tissue should not be damaged in more than in 5% of patients.

One of the possible methods of radiation therapy dose distribution in time is a continuous mode of exposure for several days. An example of this method is the brachytherapy whereby radioactive sources are implanted into the tumor or superimposed on the tumor of the skin or mucosa by means of special devices, applicators. The main advantage of this method is a sharp gradient of dose with increasing distance from the source, which allows sparing of normal tissues at adequate radiation of the tumor. The proximity of the radiation source to the object exposure is assumed here.

Some mathematical aspects of radiation therapy optimization problems are discussed in [3–10]. As noted in [7, 8], a mathematical formulation of the radiation therapy problem consists of a pair of forward and inverse problems. The inverse problem is to determine the external radiation beams, along with their locations, profiles, and intensities, which will provide a given dose distribution within the irradiated object. A significant number of mathematical models were developed for the analysis of changes in tumor volume [3–5], the calculation of optimal radiation doses [6–10], and so forth.

In contrast to the above papers, which deal with issues related to the radiation intensity optimization, we consider the geometric aspect of the inverse problem, namely, the optimal placement of radiation sources in the affected area of skin.

In this paper, like in [11], the problem of optimal planning of contact radiation therapy for cancer is considered to be a problem of optimal placing and radiation field of a finite number of sources in a continuous environment. For this task, it is necessary to place a given number of radiation sources in the affected tissue in order to provide the most homogeneous cumulative effect of sources' performance.

A mathematical model of the problem of placing of radiation sources in the affected area is proposed here, and this model is a modification of the model proposed in [11]. For the numerical solution of the problem there has been proposed and implemented a nondifferentiable optimization method, namely, the method of generalized gradient descent with space stretching towards the difference of two sequential values of gradient, Shor's r-algorithm [12, 13].

## 2. Materials and Methods

### 2.1. Some Aspects of Optimal Contact Radiation Therapy Planning

We will consider the problem of optimal location in the context of optimal planning of radiation therapy for malignant tumors. This has already been proposed in [11], where it was needed to place a given number of radiation sources in the affected tissue.

In brachytherapy, the radiation source in the tumor should be placed in such a way to provide the most homogeneous dose field, which enables the full therapeutic effect (disease-free cure tumor) to be achieved. The problem arising during radiotherapy is that in low levels exposure fields (in region of local minima field action) there could be a relapse, while in the case of high-dose radiation there could occur the necrosis, which is hard to cure.

Each cell type has its own parameters of radiosensitivity; that is, changes in the cells begin at a certain ratio of the frequency type, intensity, and duration of the radiation. In principle, any tumor can be destroyed by the influence of radiation, but healthy cells may also get damaged in this case [6, 11]. Since its inception, radiation oncology has focused on minimizing side effects. The main objective of radiation oncology is to select the optimal balance between beneficial effect of radiation and minimizing the risk of complications.

Let us consider basic techniques that are used by professionals to reduce the risk of healthy tissue damage. Firstly, the properties of healthy and cancerous cells covered by the impact must be determined as accurately as possible and, secondly, differences in radiosensitivity must also be identified. The intensity and type of radiation are selected individually for each case allowing optimizing the effectiveness of the therapy.

As many important practical optimization problems the sources locating problem, arising at the radiotherapy planning, is reduced to the problem of placing a certain number of objects in continuous environment. These objects are combined to create territorial “service fields” for “customers” that are located in this region, to minimize (or maximize) some quality criterion for the placement. Many models and approaches for solving such problems are presented in [14].

So, the considered problem may be interpreted in the following context: an affected part of the body appears as a “service field”; all cells of the affected part of the body are “customers”; and “service points” are sources of radiation that are placed inside the affected area and provide a therapeutic radiation field which inhibits destruction centers. Moreover, we assume that lesions in different parts of the body may be different. The task is to place a given number of radiation sources so that the dose field (cumulative effect of sources' performance) would be as much homogeneous as possible.

### 2.2. Constructing Mathematical Models

Let *Ω* be a limited set in Euclidean space *E* ^*n*^. Although the mathematical model of the problem of placing presented below is formulated for arbitrary finite *n*, for best interpretation we will consider the case when *n* = 2.

In contrast to [11], where the set *Ω* is homogeneous, that is, it is believed that all cells are equally affected, we will assume that there are “centers of damage,” which are points of the area in which the disease originates and is expressed in the severest way. Let us denote these centers in the following way: *θ*_*i*_ = (*θ*_*i*_^1^, *θ*_*i*_^2^,…, *θ*_*i*_^*n*^) ∈ *Ω*, $i=\bar{1,M}$. We suppose that the disease is spread from each center to neighboring cells, and the further a cell is situated from the center, the less affected it will be.

Let the influence of each center on “damage” in a point *x* ∈ *Ω* be characterized by function:

(1) $$\begin{array}{c} \rho_{i}\left(\;\left\|\;x-\theta_{i}\;\right\|\;\right)=B_{i}\exp\left(\;-\beta_{i}{\left\|\;x-\theta_{i}\;\right\|}^{2}\;\right),\;i=\bar{1,M}, \end{array}$$

where *B*_*i*_ is the damage degree of the *i*th center, *β*_*i*_ is a function parameter that shows how “wide” the ability of the *i*th center to sprawl and spread the destruction to neighboring cells is, and ‖·‖ is Euclidean norm.

Then the total degree of destruction *P*(*θ*, *x*) in a point *x* ∈ *Ω* depends on all the centers of lesions that are available in the area and is expressed by

(2) $$\begin{array}{c} P\left(\;\theta ,x\;\right)=\overset{M}{\underset{i=1}{\sum }}\rho_{i}\left(\;\left\|\;x-\theta_{i}\;\right\|\;\right). \end{array}$$

From this moment, during the solving of placement problem, we will take into account the degree of destruction in each point of the region.

**Figure 1:**
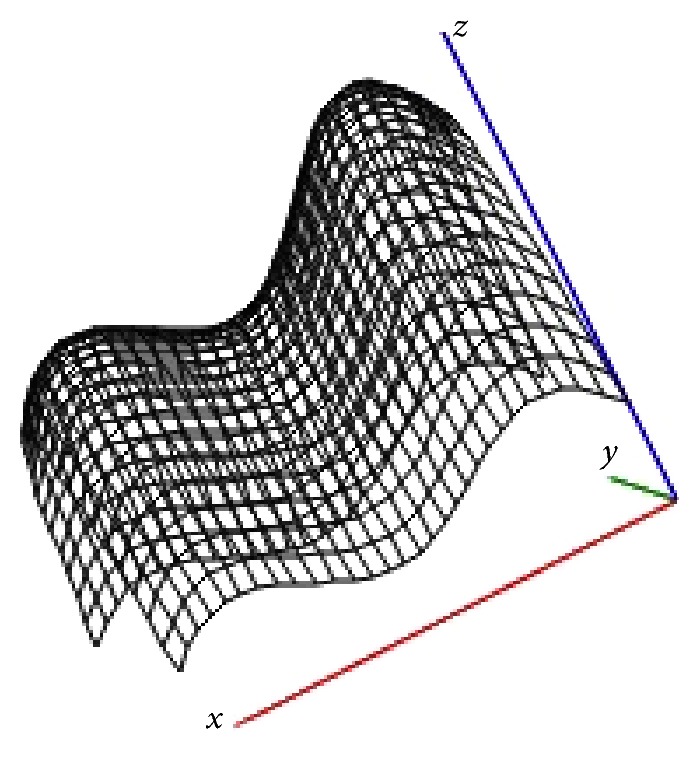
The total extent of lesions with 2 centers of damage.

**Figure 2:**
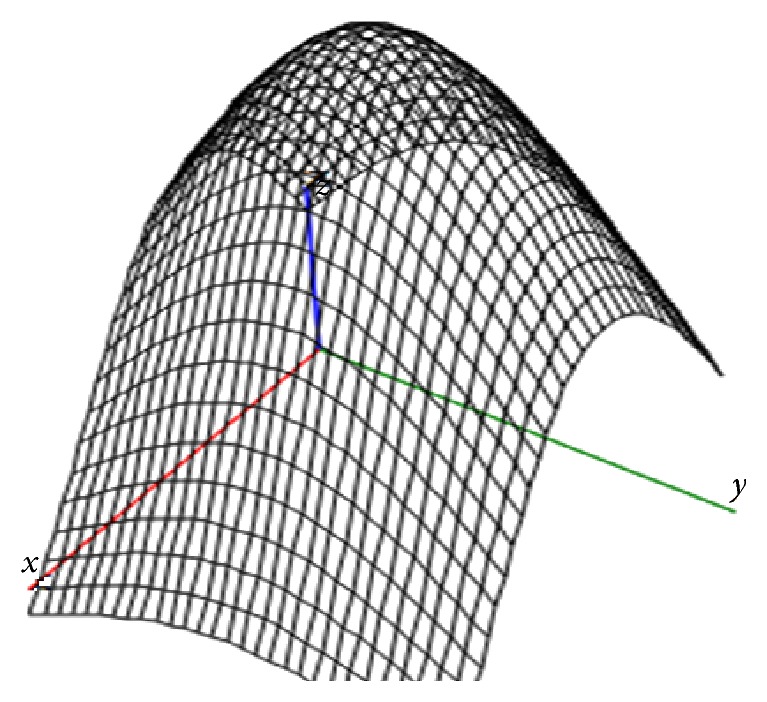
The total extent of lesions with 3 centers of damage.

Figures 1 and 2 show examples of lesions features surface (2) for a given number of centers located in the two-dimensional field *Ω* = {−2.5 ≤ *x* ≤ 2.5; −1.5 ≤ *y* ≤ 1.5} with such input data: *N* = 2; *B*_1_ = 20, *β*_1_ = 0.2; *B*_2_ = 18, *β*_2_ = 0.18 (Figure 1) and *N* = 3; *B*_1_ = 20, *β*_1_ = 0.2; *B*_2_ = 18, *β*_2_ = 0.08; *B*_3_ = 18, *β*_3_ = 0.16 (Figure 2).

Let us denote the sources of influence on the environment, which have to be placed, as *τ*_*i*_ = (*τ*_*i*_^1^, *τ*_*i*_^2^,…, *τ*_*i*_^*n*^) ∈ *Ω*, $i=\bar{1,N}$. Let the influence of each source in a point *x* ∈ *Ω* be characterized by the function:

(3) $$\begin{array}{c} d_{i}\left(\;r_{i}\;\right)=d_{i}\left(\;\left\|\;x-\tau_{i}\;\right\|\;\right),\;i=\bar{1,N}. \end{array}$$

The cumulative effect of all sources *τ*_*i*_, $i=\bar{1,N}$, in a point *x* ∈ *Ω* forms service field *D*(*τ*, *x*) that is given by

(4) $$\begin{array}{c} D\left(\;\tau ,x\;\right)=\overset{N}{\underset{i=1}{\sum }}d_{i}\left(\;\left\|\;x-\tau_{i}\;\right\|\;\right), \end{array}$$

where ‖·‖ is Euclidean norm.

We assume that the higher the damage in the point is, the closer the sources of influence should be located to this point, and hence a larger dose field in its neighborhood is needed.

The effect of all sources on a point of the affected area *Ω* may be described as follows:

(5) $$\begin{array}{c} DP\left(\;\tau ,x\;\right)=\frac{1}{P\left(\theta ,x\right)}\overset{N}{\underset{i=1}{\sum }}d_{i}\left(\;\left\|\;x-\tau_{i}\;\right\|\;\right). \end{array}$$

The problem is to place the sources *τ*_*i*_, $i=\bar{1,N}$, in *Ω* in such way, in order to maximize the minimum level of field action *DP*(*τ*, *x*) in a region under consideration (assuming that sources “clumping” is unacceptable). This problem could be mathematically formalized as follows:

(6) $$\begin{array}{c} \underset{x\in \Omega }{\min}\;DP\left(\;\tau ,x\;\right)⟶\underset{\tau \in \Omega^{N}}{max}. \end{array}$$

Note that the objective function of location optimization problem and action field of sources in continuous medium, that was considered in [11], is a special case of problem (6) when *P*(*θ*, *x*) = 1  ∀*x* ∈ *Ω*.

**Figure 3:**
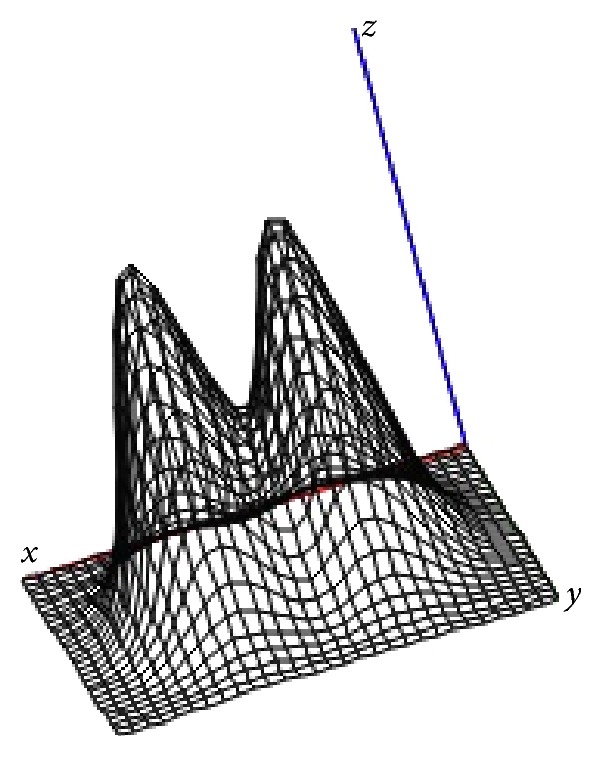
Dose field created by 2 sources: *Q* = 100, *α* = 5.

**Figure 4:**
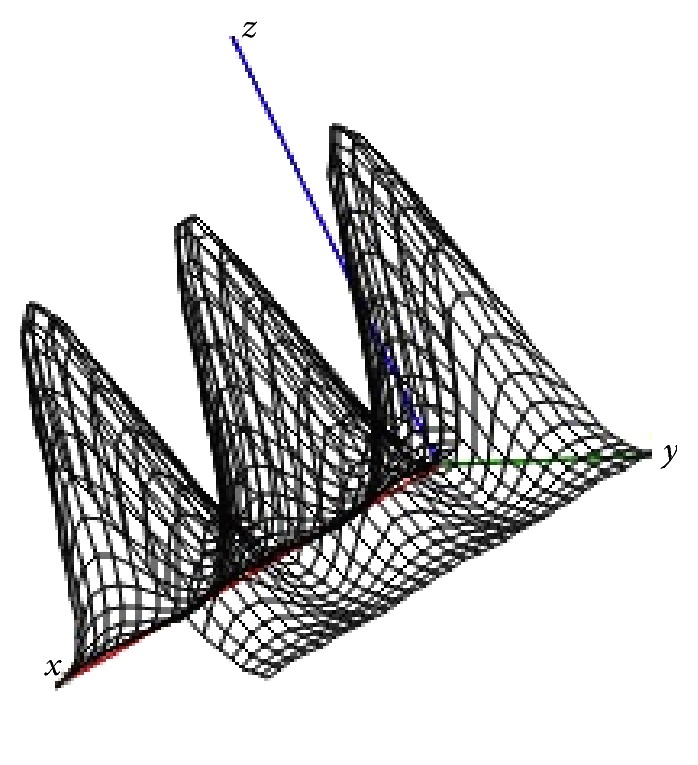
Dose field created by 3 sources: *Q* = 100, *α* = 5.

Also, unlike the mathematical model proposed in [11], where the influence of the source was described with a power function *d*_*i*_(‖*x* − *τ*_*i*_‖) = 1/‖*x* − *τ*_*i*_‖^*γ*^ (*γ* = 2), here we consider functions like

(7) $$\begin{array}{c} d_{i}\left(\;\left\|\;x-\tau_{i}\;\right\|\;\right)=Q\exp\left(\;-\alpha {\left\|\;x-\tau_{i}\;\right\|}^{2}\;\right),\;i=\bar{1,N}, \end{array}$$

where *Q* is maximum source's intensity and *α* is function parameter that shows how “wide” the impact source is. In this paper, it is suggested that these values are the same for all sources, although it is not an essential assumption. Figures 3 and 4 show surfaces of common effects functions (4) for given number of sources located in the two-dimensional region *Ω* = {−2.5 ≤ *x* ≤ 2.5; −1.5 ≤ *y* ≤ 1.5}, with condition that the impact of a single source is described by formula (7).

The choice of influence functions is based on the following reasoning. Power functions like *d*_*i*_(‖*x* − *τ*_*i*_‖) = 1/‖*x* − *τ*_*i*_‖^*γ*^, *γ* > 0, satisfying *d*_*i*_(+0) = *∞*, have a “nasty” (in computer terms) feature, causing the need to “puncture” points *x* = *τ*_*i*_ while calculating the value of the function (4) during the implementation of numerical algorithms for solving the problem. This choice of functions makes it difficult, or even impossible, to use numerical methods of maximizing which worked fine in solving nondifferentiable optimization problems and whose convergence has been theoretically proven. Function (7) does not have such deficiencies. Similar arguments were made during choosing the form of lesions functions (1). It is assumed that the parameters of influence functions and lesions functions can be determined experimentally.

Arguments in favour of the choice of the influence functions in the form of (7) are given in [15–17]. In [17], for example, the following was noted:

“… the analysis results of narrow beams photometry as well as a good agreement between the experimental data and the results of dose field calculation in a wide range of irradiation conditions have shown that the transverse component of the dosage function of point monodirectional source can be represented as a Gauss' function.”

### 2.3. Method and Algorithm for Solving

To solve this problem two methods were used: an approximate algorithm proposed in [11] and nondifferentiable optimization method, method of generalized gradient descent with stretching the space in the direction of the difference between two sequential values of the gradient (Shor's r-algorithm).

An idea of the approximate algorithm is based on the assumption that the optimal placement of sources is achieved then, and only then, when all local minima of the total action field are equal. For the numerical solution of the problem, we will organize an iterative process making, firstly, a discretization of the area. The following heuristics lies on the basis of Klepper's iterative algorithm [11]: if for some placement of sources *τ* = (*τ*_1_,…, *τ*_*N*_) function (4) reaches its global minimum in the point *x* = *z* and *τ*_*j*_ is the nearest (or one of nearest) to point *z* source, then shifting *τ*_*j*_ in the direction of point *z* (in the radial direction ${\overset{-}{\tau }}_{j}\overset{-}{z}$) by a certain fairly small amount (distance) *l* allows increasing the value of the minimum of the function (4).

For each step of the algorithm we will shift each source with a certain step *l* > 0 to the next global level of function (4), gradually decreasing the shifting step by a certain rule (*l*∶ = *ql*, 0 < *q* < 1). Iterative process is completed if either all local minimum nets are global with some precision *ε* > 0 or the step of shifting becomes less than the given minimum step.

Clearly, the objective function of problem (6) is not differentiated in the entire area *Ω*. Therefore, for solving problem (6), the method of generalized gradient descent with stretching space in the direction of the difference between two sequential values of the gradient is proposed. The effectiveness of all subgradient methods strongly depends on the conditioning of optimized functions. Therefore, to increase the speed of convergence we can try to make a coordinate transformation (change metrics) to improve conditioning. This idea is the basis of r-algorithm that combines principles of subgradient methods and variable metric methods [13]. The numerical algorithm of the method is given below.

*Algorithm*

*Initialization*. We will specify the number of sources *N* and the initial placement *τ* ^(0)^ ∈ *Ω*. Region *Ω* is covered with a rectangular grid. Further discretized region will be denoted by $\overset{-}{\Omega }$.

We calculate the value of the objective function $I(\tau^{\left(0\right)})=\min_{x\in \overset{-}{\Omega }}\;DP(\tau^{\left(0\right)},x)$ according to given initial placing sources by formula (4). Using the finite difference formulas we calculate all components of subgradient vector *g*(*τ* ^(0)^) for the objective function *I* in the point *τ* ^(0)^.

The initial test step of the r-algorithm is chosen (*h*_0_ > 0).

*The First Step*. Calculate *τ* ^(1)^ with the formula

(8) $$\begin{array}{c} \tau^{\left(1\right)}=\tau^{\left(0\right)}+h_{0}g\left(\;\tau^{\left(0\right)}\;\right). \end{array}$$

*The Second Step*. After *m* = 1,2, 3,… steps we got some placement *τ* ^(*m*)^ = (*τ*_1_^(*m*)^,…, *τ*_*N*_^(*m*)^) as a result of the algorithm. Let us describe the (*m* + 1)th step of the algorithm.

*The *(*m* + 1)*th Step*. (1) For a set *τ* ^(*m*)^ = (*τ*_1_^(*m*)^,…, *τ*_*N*_^(*m*)^) we find a value $I(\tau^{\left(m\right)})={min}_{x\in \overset{-}{\Omega }}\;DP(\tau^{\left(m\right)},x)$ from formula (4).

(2) Calculate approximate values of all components of the subgradient vector *g*(*τ* ^(*m*)^) for objective function *I* when *τ* = *τ* ^(*m*)^.

(3) Perform the (*m* + 1)th step of the r-algorithm in *H*-form; iterative formula is as follows:

(9) $$\begin{array}{c} \tau^{\left(m+1\right)}=\tau^{\left(m\right)}+h_{m}\frac{H_{m+1}g\left(\tau^{\left(m\right)}\right)}{\sqrt{H_{m+1}g\left(\tau^{\left(m\right)}\right),g\left(\tau^{\left(m\right)}\right)}}, \end{array}$$

where *H*_*m*+1_ is a matrix of space tension with coefficient *σ* (it is advisable to choose it equal to 3) in the direction of the difference between two sequential gradient values, calculated using the formula

(10) $$\begin{array}{c} H_{m+1}=H_{m}+\left(\;\frac{1}{\sigma^{2}}-1\;\right)\frac{H_{m}\xi_{m}\xi_{m}^{T}H_{m}}{\left(H_{m}\xi_{m},\xi_{m}\right)}, \end{array}$$

where *H*_0_ = *E* and *ξ*_*m*_ = *g*(*τ* ^(*m*)^) − *g*(*τ* ^(*m*−1)^).

If due to rounding in the calculations matrix *H*_*m*+1_ is not positively determined we replace it with the identity matrix.

Step *h*_*m*_ is chosen according to the condition

(11) $$\begin{array}{c} \underset{h>0}{\max}\;DP\left(\;\tau^{\left(m\right)}+h\frac{H_{m+1}g\left(\tau^{\left(m\right)}\right)}{\sqrt{H_{m+1}g\left(\tau^{\left(m\right)}\right),g\left(\tau^{\left(m\right)}\right)}}\;\right). \end{array}$$

(4) If a condition

(12) $$\begin{array}{c} \left\|\;\tau^{\left(m+1\right)}-\tau^{\left(m\right)}\;\right\|\leq \varepsilon ,\;\varepsilon >0, \end{array}$$

is not satisfied, we proceed to (*m* + 2)th step of the algorithm; otherwise, go to step  5.

(5) Consider the completion of the iterative process: the best placement is *τ* ^*∗*^ = *τ* ^(*k*)^, where *k*—iteration number at which condition (8) is performed.

This ends the algorithm.

**Note 1 (some words on the correctness of subgradient method application).**

As is well known, the notion of subgradient is introduced for convex functions. The objective function of problem (7) belongs to the class of so-called quasidifferentiable functions.

**Definition 1 (see [13]).**

The function *f*(*x*), defined on the *n*-dimensional Euclidean space *E*_*n*_, is called almost differentiable if it satisfies the following conditions: (a) any restricted area is Lipschitz (locally Lipschitz); (b) it is almost everywhere differentiable; (c) its gradient is continuous on the set *M*, where it exists.

**Definition 2 .**

A vector that is a limit point of a sequence of gradients *g*(*x*_1_), *g*(*x*_2_),…, *g*(*x*_*k*_),…, where {*x*_*k*_}_*k*=0_^*∞*^ is a sequence of points converging to a point *x*_0_ and such that at all points of the sequence function *f*(*x*) are differentiable, is called quasigradient of function *f*(*x*) in point *x*_0_.

The following theorems are accepted as true [13].

**Theorem 3 .**

Suppose that the real function *f*(*x*), defined on an open set *M* ⊂ *E*_*n*_, has finite partial derivatives in all directions: $\overset{-}{\lim_{t\to 0}}\left|\left(f(x+tv)-f(x)\right)/t\right|<+\infty$ for any *x* ∈ *M* and *v* ∈ *E*_*n*_. Then, *f*(*x*) is differentiable almost everywhere on *M*.

**Theorem 4 .**

Set *G*(*x*) of quasigradients of the quasidifferentiable function *f*(*x*) is nonempty, is bounded, and is closed in any point *x* ∈ *E*_*n*_.

**Theorem 5 .**

An arbitrary convex function *f*(*x*) is quasidifferentiable on the *n*-dimensional Euclidean space *E*_*n*_ and in point *x*_0_ any of its quasigradients coincide with some subgradient.

**Note 2 .**

It should be noted that the problem in its mathematical formulation is related to a continuous task of the ball covering. Different optimal design algorithms of ball covering a limited area presented in [18] could be applied to solving the above problem. Some of them are based on certain heuristics, and others are used as the mathematical apparatus of the Voronoi regions. Applications of the theory of continuous problems of optimal set partitioning to the problems of the single covering of bounded area of plane are described in [19].

## 3. The Results of the Computational Experiments

**Figure 5:**
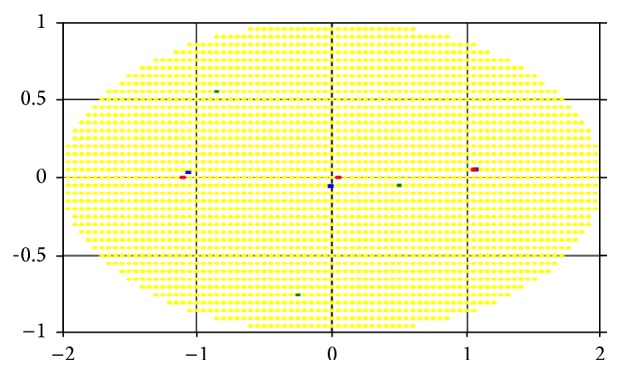
Optimal placement of 3 sources in a region with homogeneous “demand.”

**Figure 6:**
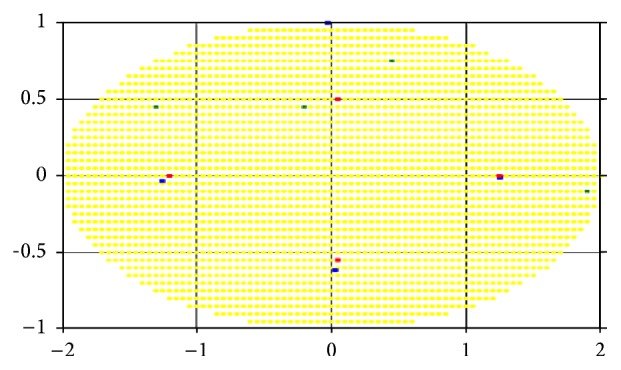
Optimal placement of 4 sources in a region with homogeneous “demand.”

**Figure 7:**
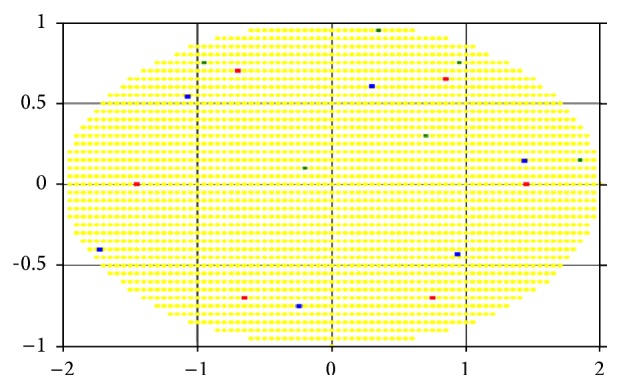
Optimal placement of 6 sources in a region with homogeneous “demand.”

Let us consider the results of the computational experiments for the two-dimensional lesion field in the shape of an ellipse example (Figures 5–7). For convenience, we take the case of a homogeneous “demand” for impact dose in area of damage, that is, when the degree of damage is the same and equal to one for every cell. Figures 5, 6, and 7 represent the optimum location of 3, 4, and 6 sources in the elliptic region, respectively. Coordinates (*x*, *y*) of radiation sources that make up the optimal solution of the problem found by r-algorithm are marked with blue squares while the optimal placement of sources obtained by approximate Klepper's algorithm [11] is marked with red squares. It should be noted that both here and in the following examples initial centers' approximation (green points on the Figure 5 and further) is the same for both algorithms.

In these, and in the following test examples, it is assumed that the radiation sources are identical and their influence function is given by formula (7), where *Q*_*i*_ = 100, *σ*_*i*_ = 2, $i=\bar{1,N}$. Parameters of Klepper's iterative algorithm are *l* = 0.8, *q* = 0.9, *l*_min_ = 0.015, *ε* = 0.01, and *h*_*x*_ = *h*_*y*_ = 0.05. Parameters of r-algorithm are *h*_0_ = 1, *σ* = 3, *ε* = 0.001, and *h*_*x*_ = *h*_*y*_ = 0.05.

Table 1 shows the comparison of the best objective functions values for the problem with homogeneity throughout the region “demand” on the degree of sources' influence obtained by the two algorithms.

As can be seen in Figures 5–7, by virtue of the fact that the area, in which sources are accommodated, is symmetric and “homogeneous” (in the sense that the “demand” on the radiation dose is the same for all points of region), the optimal placement of radiation sources is often symmetrical (as noted in [11]). However, as shown in Figures 6 and 7, the r-algorithm allows us to find an optimal solution that does not possess the symmetry property but, instead, delivers the best total dose field (see Table 1).

By comparing the results, we can conclude that in the region with homogeneous “demand” both algorithms give nearly the same results, but, increasing the number of sources, the nondifferentiable optimization algorithm gives the location with the higher objective function value. Furthermore, the r-algorithm is much faster than the Klepper's one.

**Figure 8:**
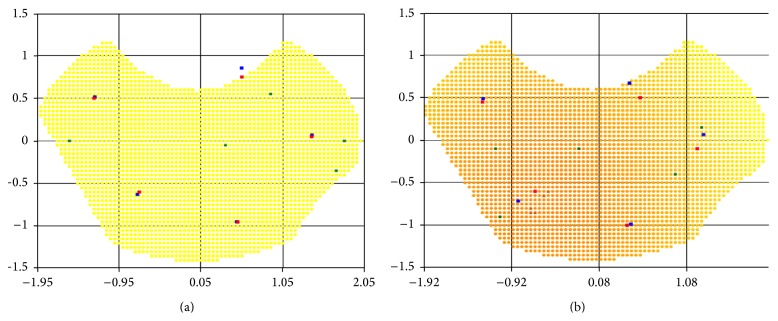
Optimal placement of 5 sources: (a) in area with homogeneous “demand”; (b) in area with inhomogeneous “demand.”

**Figure 9:**
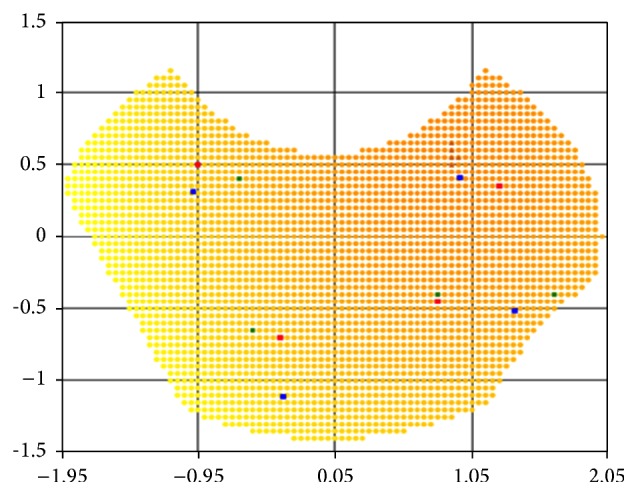
Optimal placement of 4 sources in area with inhomogeneous “demand.”

**Figure 10:**
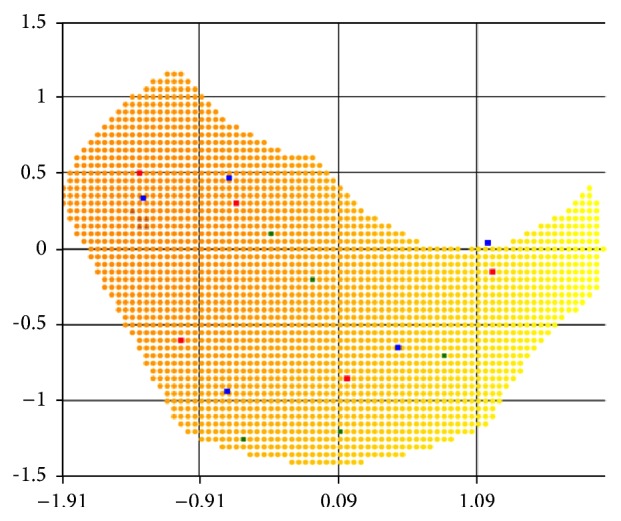
Optimal placement of 5 sources in area with the inhomogeneous “demand.”

Figures 8(b), 9, and 10 show locations found by both algorithms for 5, 4, and 5 sources, respectively, in case of inhomogeneous “demand” in the nonconvex regions. The darker the color of the point, the greater the extent of tissue damage in it and, correspondingly, the higher the “demand” on the radiation dose. For comparison, the optimum radiation sources' location for the same as in Figures 8(b) and 9 area but with unit “demand” for the entire region is represented in Figure 8(a).

Best objective functions for inhomogeneous “demand” are presented in a comparative table (Table 2).

**Note 3 .**

Table 2 lists the minimum weighted maximum total dose of radiation, the magnitude of the dose field divided by the level of the affected tissues at a point. This explains the difference in the values of hundreds of pieces of test case number 1 from the rest.

Computational experiments have allowed us to make few observations and conclusions: (1) the grinding spatial grid significantly increases the time during which the optimal (local) solution of the problem of sources' placement may be found for both algorithms; (2) both algorithms are sensitive to the choice of the initial approximation of coordinates sources to be placed and can only lead to a local problem solution; (3) if the area in which springs are accommodated has the property of symmetry and homogeneity, then the optimal arrangement of the sources will also have the symmetry property.

As one could see from the computational experiments, the results heavily depend on the initial data and the algorithm parameters, the initial approximation coordinates of centers, the step size of the spatial grid, and the step size for numerical differentiation in the evaluation of the component of the generalized gradient. In order to remove the last shortcoming, we propose to create a version of the algorithm with the elements of the theory of continuous problems of optimal set partitioning [18], namely, in calculating the components of the generalized gradient of the objective function (4) to use the Voronoi diagram constructed using the methods of OSP [19].

## 4. Conclusions

In this paper, we have proposed and analyzed a mathematical model of optimizing location and action fields of a finite number of irradiation sources in the context of radiotherapy. We have shown that, similarly to other important practical optimization problems, this problem can be reduced to the problem of placing a certain number of objects in a continuous environment. These objects are then combined to create a territorial “service field” for “customers” that are located in this region, and the problem is to minimize (or maximize) some criterion for placement. The model developed in this paper is accounting for “demand” on the value of radiation in each point of a given region, as well as a requirement of the greatest possible action field homogeneity of distributed sources.

To solve the problem of optimal distribution of irradiation sources, two different algorithms have been used: approximate Klepper's algorithm and Shor's r-algorithm. The results of the numerical experiments have shown that the use of nondifferentiable optimization techniques to solve the formulated problem is more appropriate when the demand is not homogeneous in the region under consideration.

In the future, the present model can be generalized to the case where one needs to identify not only the locations of radiation sources but also some other parameters, such as the duration, shape, and intensity of radiation. It is also envisaged to apply the theory of continuous problems of optimal set partitioning to solve problems similar to the one analyzed in this paper. The present model could be also generalized to the case of the dynamics of the irradiation process by adding the differential equation describing the change in the volume of the tumor (cancer cells) during radiotherapy. In this case, methods of solving dynamic problems of optimal sets partitioning may be found useful [20].

## Figures and Tables

**Table 1:** The results of the algorithm for a field with homogeneous “demand.”

Number of sources	The optimal objective function value obtained by r-algorithm	The optimal objective function value obtained by using Klepper's algorithm
2	21.03	21.05
3	51.52	49.871
4	91.269	89.023
5	123.245	97.055
6	171.46	157.63

**Table 2:** The results of the algorithm for a field with inhomogeneous “demand.”

Test number	Figure with optimal sources' placement	The value of the objective function (r-algorithm)	The value of the objective function (heuristic algorithm)
1	Figure 8(a)	119.262	111.786
2	Figure 8(b)	1.056	0.851
3	Figure 9	0.638	0.562
4	Figure 10	1.167	0.988
